# The Health Wagon Partners with the Virginia Department of Health to Provide COVID-19 Testing in Rural Southwest Virginia

**DOI:** 10.13023/jah.0203.12

**Published:** 2020-07-19

**Authors:** Tauna Gulley, Teresa Tyson, Ethan Collins, Rachel Helton, Paula Hill-Collins, Nicole France, Sarah Hubbard

**Affiliations:** The University of Pikeville, taunagulley@upike.edu; The Health Wagon, drtysonnp@thehealthwagon.org; The Health Wagon, ecollins@thehealthwagon.org; The Health Wagon, rhelton@thehealthwagon.org; The Health Wagon, paulahill@thehealthwagon.org

**Keywords:** Appalachia, health department, COVID-19, rural health care, mobile health clinic, health services

## Abstract

The Health Wagon has been providing care for the rural population of southwest Virginia for the past 40 years. The mission of the Health Wagon is to provide quality health care to the medically underserved people in the mountains of Appalachia. It has expanded to two stationary clinics, three mobile units, and a mobile dental unit, logging over 19,000 patients encounters in the past year.

Forty years ago, Sister Bernadette Kenny of the Catholic Order Medical Missionaries of Mary began what is now known as the Health Wagon. At that time, Sister “Bernie” traveled up the mountain roads to hollers and hilltops in her Volkswagen Beetle to provide health care to people living in the mountainous region of southwest Virginia. Today, the Health Wagon has expanded to two stationary clinics, three mobile units, and a mobile dental unit. The stationary clinics are located in Clintwood and Wise, Virginia. Before COVID, the mobile units traveled to the coal mining region of central Appalachia in southwest Virginia and provided care to residents of Wise, Lee, Scott, Dickenson, Russell, and Buchanan counties where care is provided by a licensed nurse and nurse practitioner.

In the early years, funding was provided by St. Mary’s Hospital in Norton, Virginia. However, when that hospital was bought by a large healthcare company, the Health Wagon established itself as a 501(c)3 nonprofit. The Health Wagon continues to rely on grant funding and donations from individuals, foundations, and corporations for operating costs to provide a full range of comprehensive health services and serve as a regular medical home to over 4000 patients annually in the communities where they live, so that barriers to access are lessened; last year alone more than 19,000 patient encounters were recorded.

Services include primary medical care, chest x-rays and pulmonary function testing, assistance with obtaining medications, specialty clinics, women’s health, telemedicine services, and most recently, dental services. The Health Wagon is unique as it is the oldest mobile clinic in the nation. The mission of the Health Wagon is to provide quality health care to the medically underserved people in the mountains of Appalachia. The people we care for have diabetes, hypertension, obesity, coal miner’s pneumoconiosis (black lung), and COPD, among other conditions.

We knew that patients with these underlying health conditions had increased risks for complications from COVID-19 if they contracted the virus, and we wanted to help keep them as healthy as possible during the pandemic. Our goal was to establish testing; we felt that rapid identification of an active infection would result in contact tracing, faster treatment, and better outcomes. In addition, teaching patients about ways to prevent spreading the disease to family members and others in the community could be implemented during testing.

On May 12th, during a Zoom meeting with Justin Fairfax, the Lieutenant Governor of Virginia, he asked about the health needs of the region; our response was that we needed more testing in our area and requested COVID test kits in order for the Health Wagon to have the capacity to test for COVID-19. On Friday, May 15, 500 COVID-19 test kits were delivered to the Health Wagon and the initial COVID testing team was established (Figure 1).

After receiving the kits, the Health Wagon contacted Dr. Sue Cantrell, Director, Cumberland Plateau and Lenowisco Health District Director, Virginia Department of Health, to determine where testing needs were the greatest in southwest Virginia. On May 26, in partnership with the Virginia Department of Health, the Health Wagon offered testing in Clinchco, Virginia, a small mining town in southwest Virginia.

The event was posted on social media and patients were asked to call the health department in Clintwood to be pre-screened and get an appointment for testing. Drive-through testing had four advantages: it was a fast way to test a lot of people; people who are working could schedule a break during a testing period; it helped conserve PPE (personal protective equipment); and it decreased contact between Health Wagon staff and the patients who were being tested.

On the first day of testing, an elderly lady was brought to the testing site riding an ATV driven by a neighbor, who stated the lady was 88 years of age, had been sick and needed to be tested. Both ladies were tested.

In order for us to promote health among individuals and communities during this pandemic, we must have the capacity to test. We value community outreach and collaboration. Partnering with the Health Department allowed us to obtain the laboratory services needed to obtain and report COVID-19 test results, and we are happy to partner with our elected leaders to ensure the health and safety of the communities we serve.

**Figure:**
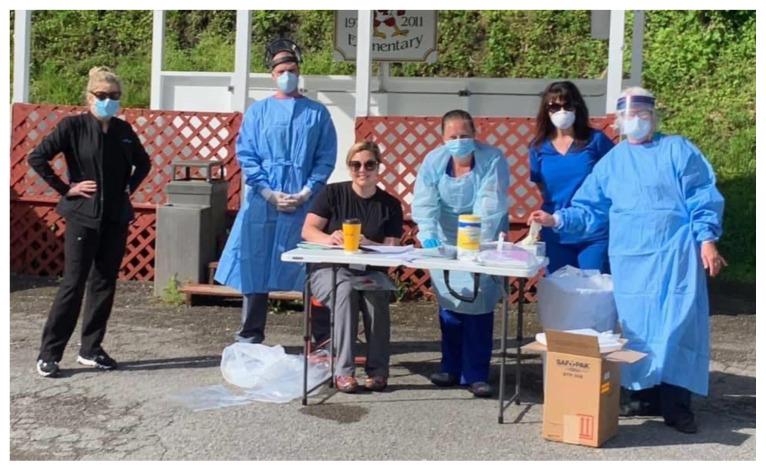
The Health Wagon COVID-19 Testing Team Teresa Tyson, Ethan Collins, Sarah Hubbard, Nicole France, Paula Hill-Collins, and Tauna Gulley (Image used with permission)

